# A Novel Triple Matrix Factorization Method for Detecting Drug-Side Effect Association Based on Kernel Target Alignment

**DOI:** 10.1155/2020/4675395

**Published:** 2020-05-28

**Authors:** Xiaoyi Guo, Wei Zhou, Yan Yu, Yijie Ding, Jijun Tang, Fei Guo

**Affiliations:** ^1^The Hemodialysis Center, The Affiliated Wuxi People's Hospital of Nanjing Medical University, 214000 Wuxi, China; ^2^School of Electronic and Information Engineering, Suzhou University of Science and Technology, Suzhou, Jiangsu 215009, China; ^3^School of Computer Science and Technology, College of Intelligence and Computing, Tianjin University, Tianjin 300072, China; ^4^Department of Computer Science and Engineering, University of South Carolina, Columbia, SC 29208, USA

## Abstract

All drugs usually have side effects, which endanger the health of patients. To identify potential side effects of drugs, biological and pharmacological experiments are done but are expensive and time-consuming. So, computation-based methods have been developed to accurately and quickly predict side effects. To predict potential associations between drugs and side effects, we propose a novel method called the Triple Matrix Factorization- (TMF-) based model. TMF is built by the biprojection matrix and latent feature of kernels, which is based on Low Rank Approximation (LRA). LRA could construct a lower rank matrix to approximate the original matrix, which not only retains the characteristics of the original matrix but also reduces the storage space and computational complexity of the data. To fuse multivariate information, multiple kernel matrices are constructed and integrated via Kernel Target Alignment-based Multiple Kernel Learning (KTA-MKL) in drug and side effect space, respectively. Compared with other methods, our model achieves better performance on three benchmark datasets. The values of the Area Under the Precision-Recall curve (AUPR) are 0.677, 0.685, and 0.680 on three datasets, respectively.

## 1. Introduction

Drug treatment of patients' diseases may be accompanied by side effects, endangering the life and health of patients. Therefore, how to quickly and accurately find potential drug side effect information becomes an important step in the drug development process. The traditional methods to detect the side effects of drugs are usually biological and pharmacological experiments. These approaches often take a long time and huge capital investment. So, it is necessary to accurately and quickly predict the potential side effects of drugs through computation-based methods [1]. Most computation-based methods for predicting drug side effects used Machine Learning (ML) classification models to predict side effect categories by extracted features from the biochemical characteristics of drugs. ML has been widely used in the field of computational biology, containing potential disease-associated microRNAs [2, 3] or circRNAs [4], O-GlcNAcylation sites [5], prediction of DNA or RNA methylcytosine sites [6, 7], protein function identification [8–12], protein remote homology [13], analyzing microbiology [14], electron transport proteins [15], drug-target interactions [16], drug-side effect association [17, 18], protein-protein interactions [19, 20], and lncRNA-miRNA interactions [21].

Pauwels and Stoven develop a predictive model of drug-side effect association by Ordinary Canonical Correlation Analysis (OCCA) and Sparse Canonical Correlation Analysis (SCCA) [1, 22]. The input feature of OCCA and SCCA is extracted from chemical structures of drugs. Cheng and Wang proposed the Phenotypic Network Inference Model (PNIM) [23] to detect new potential drug-side effect associations. Mizutani and Stoven [24] utilized cooccurrence of drug profiles and protein interaction profiles to predict side effects. The Support Vector Machine (SVM) was used to build Adverse Drug Reaction (ADR) prediction, which is based on chemical structures, biological properties of drugs, and phenotypic characteristics [25]. Zhang et al. [26–28] built an ensemble method, which was based on the Integrated Neighborhood-Based Method (INBM) and Restricted Boltzmann Machine-Based Method (RBMBM). Matrix Factorization- (MF-) based methods have been widely used for link prediction in bipartite networks of systems biology. To predict drug-target interactions, Neighborhood Regularized Logistic Matrix Factorization (NRLMF) [29], Collaborative Matrix Factorization (CMF) [30], and Graph Regularized Matrix Factorization (GRMF) [31] were developed via the MF theory.

In our study, we develop a Triple Matrix Factorization- (TMF-) based model to identify the associations of drug and side effect. TMF employs the biprojection matrix and two latent feature matrices (from drug and side effect space) to estimate the strength of new drug-side effect associations. Latent feature matrices are built via Low Rank Approximation (LRA), which could construct a lower rank matrix to approximate the original matrix. To improve the performance of prediction, Kernel Target Alignment-based Multiple Kernel Learning (KTA-MKL) is used to integrate multiple kernel matrices in drug and side effect space, respectively. Our method can fuse multivariate information (multiple kernels) and obtain new associations through matrix projection. Compared with other existing methods, our model obtains better performance on three benchmark datasets.

## 2. Method

### 2.1. Problem Description

**Figure 1:**
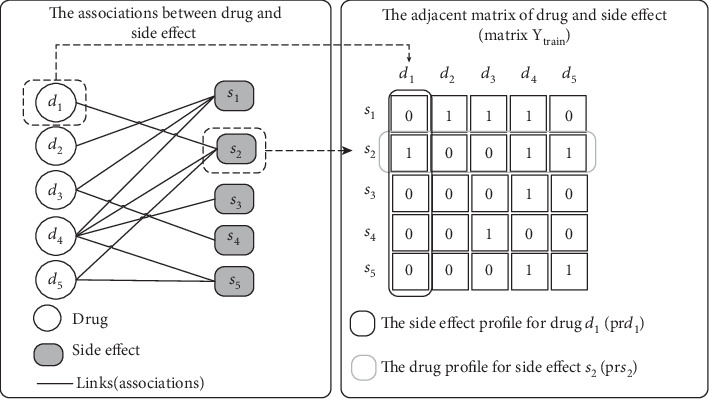
The schematic diagram of associations between drugs and side effects.

The dataset of drug-side effect association can be regarded as a bipartite network, which has *n* drugs and *m* side effects. The relationships of drug and side effect can be represented as a *n* × *m* adjacent matrix **Y** ∈ **R**^*n*×*m*^. *D* = {*d*_1_, *d*_2_, ⋯, *d*_*n*_} and *S* = {*s*_1_, *s*_2_, ⋯, *s*_*m*_} are the drug and side effect sets, respectively. *Y*_*i*,*j*_ = 1 denotes that drug *d*_*i*_ and side effect *s*_*j*_ are related; otherwise, it is 0. The associations between drugs and side effect terms are shown in Figure 1. The solid lines link the known drug-side effect associations. The hollow circles and filled squares are drugs and side effects, respectively. The prediction of new associations is a recommender task.

### 2.2. Drug Kernels and Side Effect Kernels

**Figure 2:**
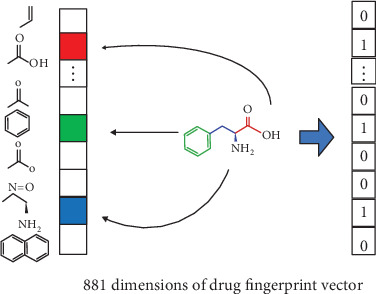
An example of the fingerprint vector.

To predict the associations of drugs and side effects, we need to construct the relationship between drugs (or side effects). In this study, we build different kernels (similarity matrices) to describe the relationships of drugs (or side effects). In drug space, the fingerprint of 881 chemical substructures is employed to encode the drug chemical structure, which is shown in Figure 2. The fingerprint represents whether some substructures are present (1) or absent (0). What is more, the known links between drugs and side effect terms (a side effect profile for a specific drug) are also used to represent the information of the subjacent network, which is shown on the right side of Figure 1. In side effect space, the drug profile for a side effect also represents the subjacent network of side effects.

There are four different types of measure functions, including Gaussian Interaction Profile (GIP) kernel [32–35], COsine Similarity (COS) [26], Correlation coefficient (Corr) [26], and Mutual Information (MI) [36, 37], which are employed to calculate the similarity between the drugs (or side effects).

For drug *d*_*i*_ and *d*_*k*_, the GIP kernel is defined as follows:

(1) $$\begin{array}{c} K_{\text{GIP}-\text{link},d}\left(d_{i},d_{k}\right)=\exp\left(-\gamma {\left\|pr_{d_{i}}-pr_{d_{k}}\right\|}^{2}\right), \end{array}$$

where *γ* is the bandwidth of the Gaussian kernel. *γ* is set as 1 in our study. **pr**_*d*_*i*__ and **pr**_*d*_*k*__ are the side effect profile of drug *d*_*i*_ and *d*_*k*_, respectively.

The COS is defined as follows:

(2) $$\begin{array}{c} K_{\text{COS}-\text{link},d}\left(d_{i},d_{k}\right)=\frac{pr_{d_{i}}pr_{d_{k}}^{T}}{\left|pr_{d_{i}}\right|\left|pr_{d_{k}}\right|}. \end{array}$$

The Corr kernel is calculated as follows:

(3) $$\begin{array}{c} K_{\text{Corr}-\text{link},d}\left(d_{i},d_{k}\right)=\frac{\text{Cov}\left(pr_{d_{i}},pr_{d_{k}}\right)}{\sqrt{\text{Var}\left(pr_{d_{i}}\right)\text{Var}\left(pr_{d_{k}}\right)}}. \end{array}$$

In order to describe the degree of correlation between two random variables, we further use Mutual Information (MI) to measure the similarity between the two random variables:

(4) $$\begin{array}{c} K_{\text{MI}-\text{link},d}\left(d_{i},d_{k}\right)=I\left(pr_{d_{i}},pr_{d_{k}}\right)=\overset{1}{\underset{u=0}{\sum }}\;\overset{1}{\underset{v=0}{\sum }}f\left(u,v\right)\log\left(\frac{f\left(u,v\right)}{f\left(u\right)f\left(v\right)}\right), \end{array}$$

where *f*(*u*) (*f*(*v*)) denotes the observed frequency of value *u* (*v*) in profile **pr**_*d*_*i*__ (**pr**_*d*_*k*__). *f*(*u*, *v*) is the observed relative frequency. Similarly, the kernels of the fingerprint (drug space: **K**_GIP−chem,*d*_, **K**_COS−chem,*d*_, **K**_Corr−chem,*d*_, and **K**_MI−chem,*d*_) and drug profile of side effects (side effect space: **K**_GIP−link,*s*_, **K**_COS−link,*s*_, **K**_Corr−link,*s*_, and **K**_MI−link,*s*_) can be constructed via the above functions. The drug space has 8 kernels, and the side effect space has 4 kernels, which are listed in Table 1.

### 2.3. Kernel Target Alignment-Based Multiple Kernel Learning

In our study, the kernel sets for drug space **K**_*d*_ = {**K**_1,*d*_, **K**_2,*d*_, ⋯, **K**_*k*_*d*_,*d*_} and side effect space **K**_*s*_ = {**K**_1,*s*_, **K**_2,*s*_, ⋯, **K**_*k*_*s*_,*s*_} are combined via multiple kernel learning, respectively. *k*_*d*_ and *k*_*s*_ are the number of kernels in drug and side effect space, respectively. A heuristic approach of Kernel Target Alignment-based Multiple Kernel Learning (KTA-MKL) [38, 39] is employed to calculate the weights of each kernel. The optimal kernels of **K**_*d*_^∗^ and **K**_*s*_^∗^ can be obtained as follows:

(5) $$\begin{array}{c} K_{d}^{*}=\overset{k_{d}}{\underset{p=1}{\sum }}\beta_{p,d}K_{p,d},\;K_{p,d}\in R^{n\times n},\\ K_{s}^{*}=\overset{k_{s}}{\underset{q=1}{\sum }}\beta_{q,s}K_{q,s},\;K_{q,s}\in R^{m\times m}, \end{array}$$

where *β*_*d*_ = {*β*_1,*d*_, *β*_2,*d*_, ⋯, *β*_*k*_*d*_,*d*_} and *β*_*s*_ = {*β*_1,*s*_, *β*_2,*s*_, ⋯, *β*_*k*_*s*_,*s*_} are the weights of kernels in drug and side effect space, respectively. KTA-MKL estimates the weight of each kernel by COsine Similarity of matrices (drug space):

(6) $$\begin{array}{c} A\left(P,Q\right)=\frac{{\langle P,Q\rangle }_{\text{F}}}{||P{||}_{\text{F}}||Q{||}_{\text{F}}}, \end{array}$$

where ${\left\|P\right\|}_{\text{F}}=\sqrt{{\langle P,P\rangle }_{\text{F}}}$ denotes the Frobenius norm. 〈**P**, **Q**〉_F_ = Trace(**P**^*T*^**Q**) is the Frobenius inner product. The value of kernel alignment can describe similarity of two kernels. KTA-MKL estimates the value between the ideal kernel matrix and the drug kernel (or side effect kernel) as follows:

(7) $$\begin{array}{c} \beta_{p,d}=\frac{A\left(K_{p,d},K_{\text{ideal},d}\right)}{\sum_{k=1}^{k_{d}}\;A\left(K_{k,d},K_{\text{ideal},d}\right)},\;p=1,2,\cdots ,k_{d},\\ \beta_{q,s}=\frac{A\left(K_{q,s},K_{\text{ideal},s}\right)}{\sum_{k=1}^{k_{s}}\;A\left(K_{k,s},K_{\text{ideal},s}\right)},\;q=1,2,\cdots ,k_{s}, \end{array}$$

where **K**_ideal,*d*_ = **Y**_train_**Y**_train_^*T*^ ∈ **R**^*n*×*n*^ and **K**_ideal,*s*_ = **Y**_train_^*T*^**Y**_train_ ∈ **R**^*m*×*m*^ are the ideal kernels of drug and side effect, respectively, which are built via the training label (known associations).

### 2.4. Triple Matrix Factorization-Based Model

Inspired by MF [29–31, 40], the similarity between drugs (or side effects) can be approximated by the inner product of two drug (or side effect) features as follows:

(8) $$\begin{array}{c} K_{d}^{*}\approx AA^{T},\;A\in R^{n\times r_{d}},\\ K_{s}^{*}\approx BB^{T},\;B\in R^{m\times r_{s}}, \end{array}$$

where **A** and **B** are the matrices of Low Rank Approximation and *r*_*d*_ and *r*_*s*_ are the dimensions of the latent feature space in drug and side effect space, respectively. The objective function of TMF is defined as follows:

(9) $$\begin{array}{c} \min\;J\left(\Theta \right)={\left\|Y_{\text{train}}-A\Theta B^{T}\right\|}_{\text{F}}^{2}+\lambda {\left\|\Theta \right\|}_{\text{F}}^{2}, \end{array}$$

where Θ ∈ **R**^*r*_*d*_×*r*_*s*_^ is the biprojection matrix. *λ* is the regularization coefficient of Θ. In our study, *λ* is set as 1.

Let *∂J*/*∂*Θ = 0, so we can obtain functions as follows:

(10) $$\begin{array}{c} \frac{\partial \left({\left\|Y_{\text{train}}-A\Theta B^{T}\right\|}_{\text{F}}^{2}+\lambda {\left\|\Theta \right\|}_{\text{F}}^{2}\right)}{\partial \Theta }=0, \end{array}$$

(11) $$\begin{array}{c} -2A^{T}\left(Y_{\text{train}}-A\Theta B^{T}\right)B+2\lambda \Theta =0, \end{array}$$

(12) $$\begin{array}{c} A^{T}A\Theta B^{T}B+\lambda \Theta =A^{T}Y_{\text{train}}B, \end{array}$$

(13) $$\begin{array}{c} A^{T}A\Theta +\lambda \Theta {\left(B^{T}B\right)}^{-1}=A^{T}Y_{\text{train}}B{\left(B^{T}B\right)}^{-1}, \end{array}$$

(14) $$\begin{array}{c} A^{T}A\Theta +\lambda \Theta {\left(B^{T}B\right)}^{-1}=A^{T}Y_{\text{train}}{\left(B^{T}\right)}^{-1}, \end{array}$$

where Equation (14) is a Sylvester equation. The final prediction can be constructed by

(15) $$\begin{array}{c} Y^{*}=A\Theta B^{T}. \end{array}$$

**Figure 3:**
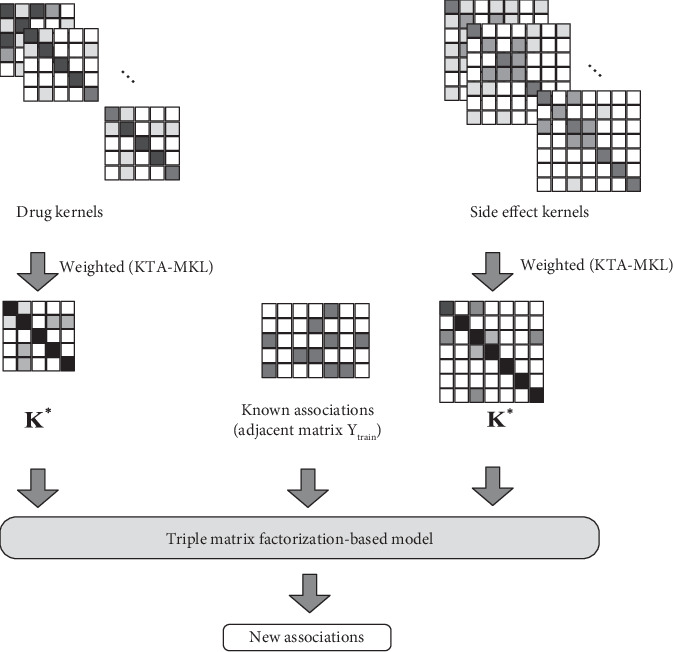
Overview of our method.

The overview of our proposed method is shown in Figure 3 and Algorithm 1.

## 3. Result

In this section, we employed benchmark dataset to evaluate our approach and compared it with other existing methods.

### 3.1. Datasets

In order to test the performance of our model, three types of datasets are employed in our study. They are Pauwels's dataset, Liu's dataset, and Mizutani's dataset, which are collected from the DrugBank [41], SIDe Effect Resource (SIDER) [42], KEGG DRUG [43], and PubChemCompound [44, 45].  Table 2 lists benchmark datasets of this study.

### 3.2. Evaluation Measurements

The training adjacent matrix can be obtained via randomly setting known associations as 0. In this study, we use 5-fold Cross-Validation (5-CV) and 5-fold local Cross-Validation (5 local CV) to test our method. 5-CV randomly sets known associations as 0 in the whole matrix. 5 local CV is employed to evaluate the prediction of new drugs, which do not have any side effect information. 5 local CV sets some rows of the adjacent matrix as 0 to test related drugs. The Area Under the Precision-Recall curve (AUPR) and Area Under the receiver operating Characteristic curve (AUC) are utilized to evaluate the performance of prediction.

### 3.3. Selecting Optimal Parameters

**Figure 4:**
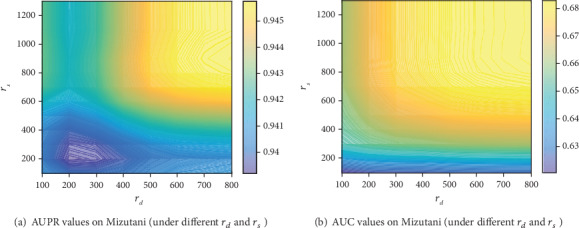
The AUC and AUPR values (under different *r*_*d*_ and *r*_*s*_).

In this section, we use the grid search method to get the optimal *r*_*d*_ and *r*_*s*_. We test different values of and from 100 to the max value with the step of 100. The results of the grid search method are shown in Figure 4 (on Mizutani's dataset by 5-CV). *r*_*d*_ = 700 and *r*_*s*_ = 800 are the best parameters (AUPR) on Mizutani's dataset. In Figure 4, the lower value of AURP and AUC is blue, and the higher value is yellow. On the other two datasets, we use the same parameters of *r*_*d*_ and *r*_*s*_.

### 3.4. Performance of Different Kernels

**Figure 5:**
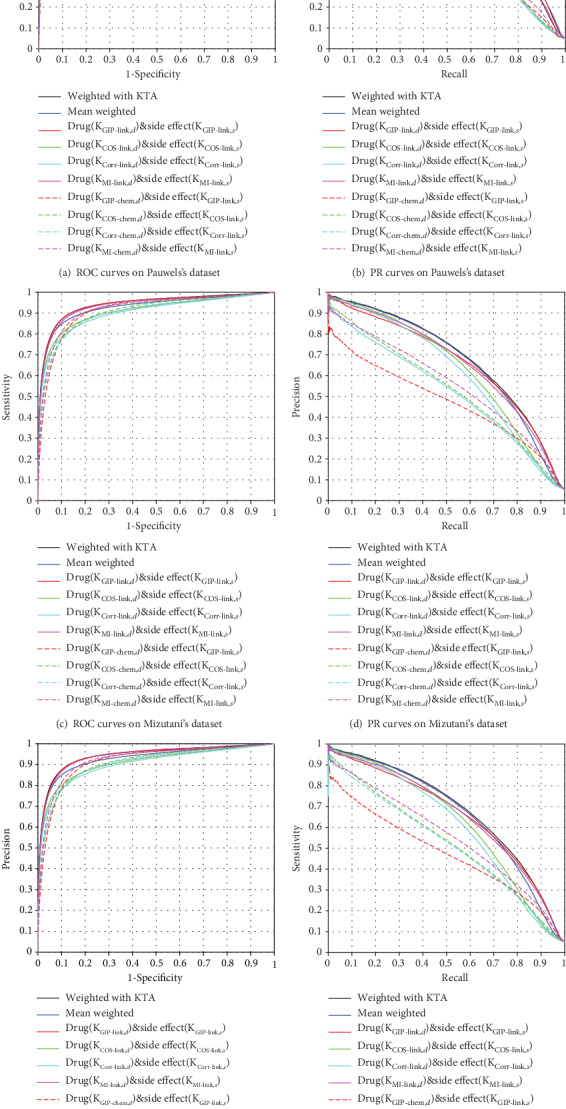
The ROC and PR curves of different models (single kernel and multiple kernels).

We evaluate the performance of multiple kernels and single kernel on three datasets. The results of prediction are listed in Table 3 and Figure 5. Obviously, the kernels of **K**_MI−link,*d*_ and **K**_MI−link,*s*_ have better performance on Pauwels's dataset (AUPR: 0.6557, AUC: 0.9079), Mizutani's dataset (AUPR: 0.6615, AUC: 0.9369), and Liu's dataset (AUPR: 0.6587, AUC: 0.9408). In addition, the KTA-MKL model achieves the best results on Pauwels's dataset (AUPR: 0.6765, AUC: 0.9434), Mizutani's dataset (AUPR: 0.6847, AUC: 0.9409), and Liu's dataset (AUPR: 0.6801, AUC: 0.9426), respectively. KTA-MKL could combine kernels from different sources via the heuristic method, which is better mean weighted.

In Table 4, we list the weight of each kernel on three datasets. We can find that the weights of **K**_MI−link,*d*_ and **K**_MI−link,*s*_ are the highest than other kernels. At the same time, their performance is also the best. KTA-MKL could reduce bias of kernels by the low weights.

### 3.5. Comparison with Existing Methods

To evaluate the performance of the TMF model, we compare it with other methods. The results are listed in Table 5. Obviously, our method (TMF) achieves the best results on Pauwels's dataset (AUPR: 0.677), Mizutani's dataset (AUPR: 0.685), and Liu's dataset (AUPR: 0.680). Zhang et al.'s work (ensemble model) [26] obtained the good performance of AUPRs (0.660, 0.666, and 0.661). The best AUCs (0.954, 0.950, and 0.953) are achieved by Neighborhood Regularized Logistic Matrix Factorization (NRLMF) [29], which is also based on Matrix Factorization (MF). The results of other MF-based models, including Collaborative Matrix Factorization (CMF) [30] and Graph Regularized Matrix Factorization (GRMF) [31], are competitive. Local and Global Consistency (LGC) [18] is our previous work. LGC obtains the second best results of AUPR (0.668, 0.673, and 0.670) on three datasets, respectively.

### 3.6. Local CV and Case Study

**Figure 6:**
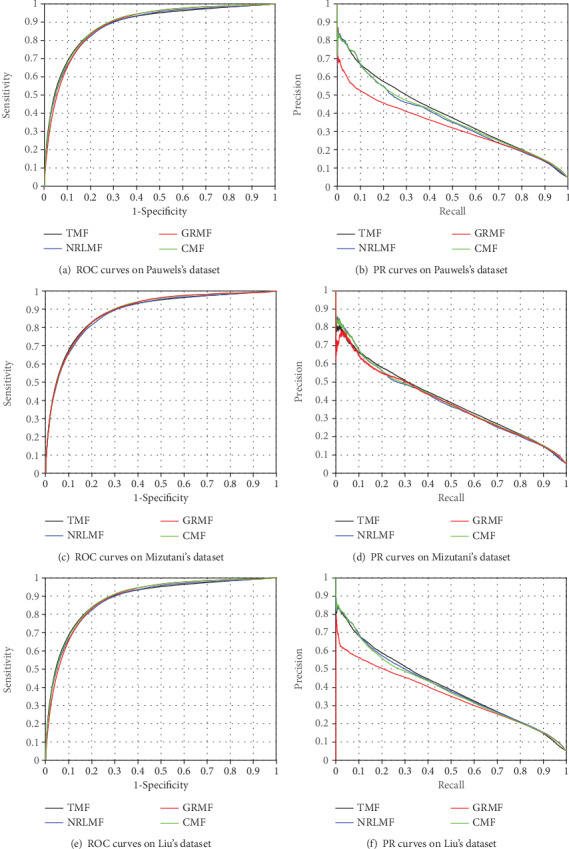
The ROC and PR curves of different methods via 5 local CV.

In some cases, certain drugs are new and have no information of side effects. The 5 local CV is employed to test the performance of the side effect prediction for new drugs. In this section, we also compare TMF with other MF-based models, including NRLMF, CMF, and GRMF. The results are listed in Table 6 and Figure 6.

The proposed method (TMF) achieves the best results of AUPRs on Pauwels's dataset (AUPR: 0.392), Mizutani's dataset (AUPR: 0.399), and Liu's dataset (AUPR: 0.401). Other MF-based models also are still comparable with our results. NRLMF obtains AUPRs of 0.374, 0.390, and 0.398 on three datasets, respectively.

To predict the side effects of a new drug, our model calculates the strength of associations between the new drug and all existing side effects. The predictive strength scores of TMF will be ranked by descending order. The higher the value of the score, the higher the possibility of associations. In this section, we discuss two cases (drug caffeine and captopril on Mizutani's dataset) of top 10 associations predicted. The details are listed in Tables 7 and 8. Results are checked by the masked associations between drug caffeine (or captopril) and side effects.

### 3.7. Running Time

We evaluate the performance for predictive models of running time. The results of test are listed in Table 9. The running time of CMF is less than our method (TMF), LGC, GRMF, and NRLMF on Pauwels's dataset (910 seconds), Mizutani's dataset (757 seconds), and Liu's dataset (846 seconds). TMF costs 977, 873, and 929 seconds, which are less than the ensemble model [26].

## 4. Conclusion and Discussion

In this study, we develop a Triple Matrix Factorization-based model to predict the associations between drugs and side effect terms. In drug space, several kernels are constructed from the chemical substructure fingerprint and known side effect-associated subnet. The side effect kernels are built from the known drug-associated subnet. The kernel functions include GIP, COS, Corr, and MI. Above kernels are combined by KTA-MKL in drug and side effect space, respectively. The integrated kernel matrices (including drug and side effect) are Low Rank Approximation in the TMF model. Our model (TMF) is tested on three benchmark datasets of drug-side effect association. Compared with other excellent methods, TMF achieves the best results (5-CV) on Pauwels's dataset (AUPR: 0.677), Mizutani's dataset (AUPR: 0.685), and Liu's dataset (AUPR: 0.680), respectively. In addition, our model is also compared with CMF, GRMF, and NRLMF under 5 local CV. The best AUPRs are achieved on Pauwels's dataset (AUPR: 0.392), Mizutani's dataset (AUPR: 0.399), and Liu's dataset (AUPR: 0.401). However, our method does not consider the topological relationship of drugs or side effects. In the future, a graph- or hypergraph-embedded MF-based model will be developed to improve the predictive performance of drug-side effect association.

## Figures and Tables

**Algorithm 1:**
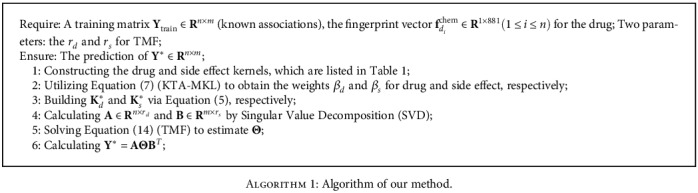
Algorithm of our method.

**Table 1:** Summary of kernels for two feature spaces.

	Chemical fingerprint (drug space)	Side effect profiles (drug space)	Drug profiles (side effect space)
GIP	**K**_GIP−chem,*d*_	**K**_GIP−link,*d*_	**K**_GIP−link,*s*_
COS	**K**_COS−chem,*d*_	**K**_COS−link,*d*_	**K**_COS−link,*s*_
Corr	**K**_Corr−chem,*d*_	**K**_Corr−link,*d*_	**K**_Corr−link,*s*_
MI	**K**_MI−chem,*d*_	**K**_MI−link,*d*_	**K**_MI−link,*s*_

**Table 2:** Three benchmark datasets.

Datasets	Drugs	Side effects	Associations
Pauwels's dataset	888	1385	61,102
Mizutani's dataset	658	1339	49,051
Liu's dataset	832	1385	59,205

**Table 3:** The performance of different kernels via 5-fold Cross-Validation.

Models	Pauwels's dataset		Mizutani's dataset		Liu's dataset	
	AUPR	AUC	AUPR	AUC	AUPR	AUC
**K**_GIP−chem,*d*_ & **K**_GIP−link,*s*_^a^	0.4420	0.8950	0.4735	0.9148	0.4718	0.9145
**K**_COS−chem,*d*_ & **K**_COS−link,*s*_^a^	0.4892	0.8994	0.5343	0.9070	0.5224	0.9067
**K**_Corr−chem,*d*_ & **K**_Corr−link,*s*_^a^	0.4994	0.8981	0.5217	0.9005	0.5143	0.9026
**K**_MI−chem,*d*_ & **K**_MI−link,*s*_^a^	0.4978	0.9079	0.5591	0.9214	0.5529	0.9238
**K**_GIP−link,*d*_ & **K**_GIP−link,*s*_^b^	0.6254	0.9300	0.6623	0.9376	0.6574	0.9398
**K**_COS−link,*d*_ & **K**_COS−link,*s*_^b^	0.5861	0.9035	0.6324	0.9090	0.6252	0.9087
**K**_Corr−link,*d*_ & **K**_Corr−link,*s*_^b^	0.5833	0.8999	0.6123	0.9014	0.6047	0.9013
**K**_MI−link,*d*_ & **K**_MI−link,*s*_^b^	0.6557	0.9428	0.6615	0.9369	0.6587	0.9408
Mean weighted^c^	0.6598	0.9353	0.6724	0.9280	0.6651	0.9285
KTA-MKL^c^	0.6765	0.9434	0.6847	0.9409	0.6801	0.9426

^a^The TMF uses the drug fingerprint and drug profile for side effects. ^b^The TMF uses the side effect profile for drugs and drug profile for side effects. ^c^The TMF uses the drug fingerprint, side effect profile for drugs, and drug profile for side effects.

**Table 4:** The kernel weights on three datasets.

Kernel	Pauwels's dataset	Mizutani's dataset	Liu's dataset
**K**_GIP−chem,*d*_	0.1159	0.1168	0.1167
**K**_COS−chem,*d*_	0.1224	0.1226	0.1226
**K**_Corr−chem,*d*_	0.1200	0.1203	0.1203
**K**_MI−chem,*d*_	0.1113	0.1122	0.1116
**K**_GIP−link,*d*_	0.0596	0.0621	0.0613
**K**_COS−link,*d*_	0.1538	0.1533	0.1528
**K**_Corr−link,*d*_	0.1507	0.1498	0.1497
**K**_MI−link,*d*_	0.1664	0.1628	0.1650
**K**_GIP−link,*s*_	0.0151	0.0173	0.0152
**K**_COS−link,*s*_	0.3286	0.3374	0.3380
**K**_Corr−link,*s*_	0.2909	0.2865	0.2855
**K**_MI−link,*s*_	0.3654	0.3588	0.3613

**Table 5:** Comparison to existing methods via 5-fold Cross-Validation.

Datasets	Methods	AUPR	AUC
Pauwels	Pauwels's method^a^	0.389 ± N/A	0.897 ± N/A
	Liu's method^a^	0.345 ± N/A	0.920 ± N/A
	Cheng's method^a^	0.588 ± N/A	0.922 ± N/A
	RBMBM^a^ [26]	0.612 ± N/A	0.941 ± N/A
	INBM^a^ [26]	0.641 ± N/A	0.934 ± N/A
	Ensemble model^a^ [26]	0.660 ± N/A	0.949 ± N/A
	CMF^b^	0.646 ± 0.007	0.939 ± 0.005
	GRMF^b^	0.643 ± 0.006	0.937 ± 0.005
	NRLMF^b^	0.654 ± 0.005	0.954 ± 0.005
	LGC^b^	0.668 ± 0.008	0.952 ± 0.007
	Our method	0.677 ± 0.004	0.943 ± 0.003

Mizutani	Mizutani's method^a^	0.412 ± N/A	0.890 ± N/A
	Liu's method^a^	0.366 ± N/A	0.918 ± N/A
	Cheng's method^a^	0.599 ± N/A	0.923 ± N/A
	RBMBM^a^ [26]	0.619 ± N/A	0.939 ± N/A
	INBM^a^ [26]	0.646 ± N/A	0.932 ± N/A
	Ensemble model^a^ [26]	0.666 ± N/A	0.946 ± N/A
	CMF^b^	0.645 ± 0.005	0.938 ± 0.006
	GRMF^b^	0.646 ± 0.007	0.937 ± 0.007
	NRLMF^b^	0.660 ± 0.006	0.950 ± 0.005
	LGC^b^	0.673 ± 0.007	0.948 ± 0.007
	Our method	0.685 ± 0.006	0.941 ± 0.008

Liu	Liu's method^a^	0.278 ± N/A	0.907 ± N/A
	Cheng's method^a^	0.592 ± N/A	0.922 ± N/A
	RBMBM^a^ [26]	0.616 ± N/A	0.941 ± N/A
	INBM^a^ [26]	0.641 ± N/A	0.934 ± N/A
	Ensemble model^a^ [26]	0.661 ± N/A	0.948 ± N/A
	CMF^b^	0.649 ± 0.006	0.938 ± 0.005
	GRMF^b^	0.650 ± 0.007	0.938 ± 0.008
	NRLMF^b^	0.656 ± 0.005	0.953 ± 0.006
	LGC^b^	0.670 ± 0.008	0.951 ± 0.007
	Our method	0.680 ± 0.005	0.943 ± 0.006

^a^Results are derived from [26]. ^b^Results are derived from [18].

**Table 6:** Comparison with MF-based models via 5-fold local Cross-Validation.

Datasets	Methods	AUPR	AUC
Pauwels	CMF^∗^	0.382 ± 0.006	0.894 ± 0.004
	GRMF^∗^	0.358 ± 0.008	0.883 ± 0.005
	NRLMF^∗^	0.374 ± 0.007	0.886 ± 0.004
	Our method	0.392 ± 0.008	0.889 ± 0.004

Mizutani	CMF^∗^	0.395 ± 0.005	0.889 ± 0.004
	GRMF^∗^	0.392 ± 0.008	0.890 ± 0.006
	NRLMF^∗^	0.390 ± 0.006	0.882 ± 0.005
	Our method	0.399 ± 0.013	0.886 ± 0.003

Liu	CMF^∗^	0.393 ± 0.007	0.894 ± 0.005
	GRMF^∗^	0.379 ± 0.008	0.895 ± 0.006
	NRLMF^∗^	0.398 ± 0.006	0.897 ± 0.004
	Our method	0.401 ± 0.015	0.891 ± 0.004

^∗^Results are derived from [18].

**Table 7:** Top 10 ranks of predictive side effects for drug caffeine.

Side effect	Score	Ranks	Confirmed
Diarrhea	0.3992	1	Yes
Diabetic neuropathy	0.3893	2	Yes
Varicocele	0.3844	3	Yes
Gynecomastia	0.3815	4	Yes
Conjunctivitis	0.3794	5	Yes
Telangiectasia	0.3737	6	No
Lump	0.3663	7	Yes
Dyskinesia	0.3638	8	No
Palpitations	0.3632	9	No
Fecal incontinence	0.3563	10	Yes

**Table 8:** Top 10 ranks of predictive side effects for drug captopril.

Side effect	Score	Ranks	Confirmed
Diarrhea	0.4150	1	No
Diabetic neuropathy	0.4043	2	Yes
Varicocele	0.4004	3	Yes
Conjunctivitis	0.3973	4	Yes
Gynecomastia	0.3938	5	Yes
Myoglobinuria	0.3885	6	No
Esophageal varices	0.3854	7	Yes
Lump	0.3806	8	Yes
Palpitations	0.3770	9	No
Eclampsia	0.3674	10	Yes

**Table 9:** The running time (seconds) via 5-fold Cross-Validation.

Model	Pauwels	Mizutani	Liu
Our method	977	873	929
LGC [18]	1290	1170	1211
CMF [18]	910	757	846
GRMF [18]	1360	1175	1282
NRLMF [18]	1966	1250	1911
Ensemble model [26]	4330	2715	3611

## Data Availability

The datasets, codes and corresponding results are available at https://figshare.com/s/10ee9c07123304a0ef82.
